# Story of the Insane from Year to Year

**Published:** 1894-07-14

**Authors:** 


### The Institutional Workshop.

### THE STORY OF THE INSANE FROM YEAR TO YEAR.

(Seventh Series.)

### ENNIS DISTRICT ASYLUM.

The accommodation in this asylum is given at 380 beds, and it contained 370 beds on the last day of 1893. The admissions were 99, the recoveries 38, and the deaths 23. Thrown into percentages these numbers show that the recoveries were 38·4 per cent., and the deaths 6·3. The former is about the average, and the latter considerably below it; that is, the average for England. Dr. Gelston is right in stating his opinion that the wards of a workhouse are not proper places for persons of unsound mind, and he thinks the 18 cases now in the Ennis Workhouse should be transferred to the asylum. We learn, with some surprise, that there is no head attendant, and we wonder who performs the responsible duties usually entrusted to that official. There is a head nurse, whose unremitting energy Dr. Gelston mentions, and she deserves the praise, as she has been able to induce 84 per cent. of the female patients to employ themselves. On the alleged increase of insanity in Ireland, Dr. Gelston sums up pretty much as follows, but he is careful to state that it is the increase in the asylum population he is referring to. First. The prejudice against asylums has diminished, and cases formerly treated at home are now certified and removed to asylums. Second. Consanguineous marriages, these being more frequent along the sea coast. Third. Excessive use of alcohol, and more especially of bad alcohol. Fourth. The disturbed state of the county. The Commissioners say that the medical superintendent shows a zealous and intelligent interest in the working of the asylum. The cost per head per annum is £23 5s.

### THE RETREAT, YORK.

There were 151 patients in residence, being an increase of one only during the year. The admissions were 27, the recoveries 8, and the deaths 7.

Of the patients at present in the asylum, 77 are members of the Society of Friends, 11 are closely connected with the society, and 63 have no connection. The average weekly cost of maintenance is 38s. 2¼d., and the average weekly payment per patient is a few shillings over the average cost. We have often regretted the omission from the reports of similar institutions of a table showing the various sums paid by patients.

During the year Dr. Baker, who had been Medical Superintendent for eighteen years, resigned the office, and was succeeded by Dr. Bedford Pierce.

### ROYAL ASYLUM, ABERDEEN.

Here the numbers went up during the year from 664 to 686. The admissions were 202, the recoveries 79, and the deaths 49. The recoveries stand at 39·1 per cent., and the deaths at 7·2 per cent. Hereditary influence accounts for 69 of the admissions and drink for 31, so that these two causes have to be credited with producing nearly 50 per cent. of the insanity of Aberdeenshire. Of the deaths 18 were owing to brain diseases and 6 to consumption. One of the patients died of influenza at the age of 81, and he had been an inmate for 64 years. Most likely he was a pauper, and his maintenance must have cost the county a nice little sum; but if he were a private patient his death must have disturbed Dr. Reid's calculations, for he could hardly avoid looking on him as a source of fixed income for the asylum. Dr. Angus, the Senior Assistant Medical Officer, is doing good work when he gives instructions to the attendants in nursing. He will find that the time bestowed on this will bear fruit in many ways, and that his own work in the wards will be made easier by the more intelligent manner in which his orders are carried out. Dr. Reid laments the fact that we speak of "Lunatic Asylums" and not of "Hospitals for Diseases of the Nervous System"; and he thinks the latter term would be more pleasing to those who have relatives in the institution. Perhaps it would. There is much in a name, and as our ears are very easily shocked in this last decade of the nineteenth century, it is a pity they should be offended by the ugly word "lunatic," although the derivation is not suggestive of ugliness, and we hope Dr. Reid will initiate the change, a change we shall be pleased to herald and support. The Commissioners say "the asylum is managed with great ability and conscientious painstaking"; and the committee say "the work of the institution has been conducted throughout with care and efficiency." Private patients pay from £30 to £50 a year, and those who wish special accommodation go up to £250.

### ST. ANDREW'S HOSPITAL, NORTHAMPTON.

On January 1st, 1893, this asylum contained 324 patients, and by December 31st the numbers had risen to 343. There were 81 admissions, 27 recoveries, and 12 deaths. Intemperance in drink is given as the cause of insanity in only two of the admissions, while hereditary influence accounts for 14, and previous attacks for 16. In 17 cases there was no cause stated. The recoveries, calculated on the admissions, stand at 33·3 per cent., and the deaths at 3·6 on the average number resident. The latter is extremely low, and the recovery rate would rise to 40·90 per cent. if transfers were excluded in the calculations. The Commissioners say that the hospital and its dependencies "are all generally in very good order," and the committee "express their thanks to the medical superintendent and the officers of the institution for the pains and trouble they have taken in the administration of affairs." On page 32 of the report there is a very useful and instructive table showing the number of patients who have received charitable aid during the year. Eight patients were kept free of charge and 13 at or under 10s. a week.

### LONDONDERRY DISTRICT ASYLUM.

This asylum is intended to accommodate 344 patients, and it began the year with 427 patients and ended with 424, so there must have been considerable overcrowding. There were 109 admissions, 40 recoveries, and 51 deaths. The recoveries work out at 36·69 per cent, and the deaths at 11·99. Drink is the assigned cause in 12 instances, and hereditary influence only 8; but as 58 cases are returned as "unknown" it is manifest that this table is useless. Of the deaths 11 were caused by brain diseases, 14 by consumption, and 17 by inflammation of the lungs. The death-rate from consumption is high, and that from pneumonia is, so far as our asylum experience goes, unprecedented. Dr. Hetherington does not give any explanation of it, and we can only surmise that the asylum was visited by an epidemic form of pleuro-pneumonia. Classes for the instruction of the attendants are held by Dr. Ruttledge, and it is to be hoped that several of the staff "will qualify as trained attendants for mental diseases"

The Committee of Grand Jury report that they "are highly satisfied with the cleanliness and good order of every part of the building," and the Commissioners say that the "records show that the medical staff are alive to the necessity of keeping pace with the study of psychological medicine now being carried out in many of our public asylums." The average cost per head per annum is £23 4s. 5d.

